# Attachment, loneliness, and social support as moderators of conversational AI–based mental health outcomes

**DOI:** 10.1038/s41746-026-02974-y

**Published:** 2026-07-07

**Authors:** Anat Shoshani, Bar Gurfinkel, Ariel Kor, Or Kanarek, Romi Segev, Or Shafir, Romi Arbel, Yael Ben-Haim

**Affiliations:** 1 Baruch Ivcher School of Psychology, Reichman University, Herzliya, Israel; 2 Faculty of Medicine, The Hebrew University of Jerusalem, Jerusalem, Israel; 3 School of Medicine, Yale University, New Haven, CT USA

**Keywords:** Health care, Psychology, Psychology

## Abstract

Conversational AI is emerging as a scalable tool for psychological support, yet its clinical effectiveness and the relational factors underlying user benefit remain underexplored. This pre-registered randomized controlled trial (*N* = 977 university students; ages 18–32) compared an AI-based conversational intervention, integrative group therapy, and a waitlist control over 12 weeks, with a 3-month follow-up. The AI intervention produced greater reductions in generalized anxiety symptoms and stronger gains in well-being and life satisfaction than both comparators at post-intervention and follow-up and reduced depression relative to control. Group therapy improved depression at post-intervention and enhanced well-being and life satisfaction, with well-being maintained at follow-up. PTSD symptoms showed no group differences. Relational factors, including loneliness, low perceived social support, and insecure attachment, predicted greater improvement and higher engagement with the AI system, particularly among participants with anxious or avoidant attachment. Participants with high loneliness engaged approximately twice as much with the AI intervention as those with low loneliness, and the association between relational vulnerability and reductions in generalized anxiety symptoms was fully mediated by engagement. Findings suggest that adaptive conversational AI can complement traditional care while addressing relational vulnerability.

## Introduction

Over the past decade, the global mental health crisis has sharply intensified, with demand for care far exceeding the availability of trained professionals^1^. Simultaneously, help-seeking behaviors are shifting. Large-scale surveys reveal that conversational AI systems are now used not only for entertainment or technical support but also for sensitive personal issues such as life planning, relationships, and mental health. For instance, a 2025 Deloitte survey reported that 85% of generative AI users applied these systems for personal purposes, many engaging in discussions about relationships or emotional well-being and even interacting with chatbots as companions or counselors^2^. This growing turn toward AI for intimate, human-like consultation underscores an expanding public openness to digital companionship in domains once reserved for close others or professionals.

This growing reliance on AI for personal guidance has intensified interest in its role in mental health care. While enthusiasm for AI-based conversational agents is increasing, their place in clinical settings remains contested and not yet fully established. Early evidence indicates that these systems can simulate empathic dialogue, deliver evidence-based strategies, and provide immediate, personalized responses at any time^3,4^. Meta-analyses and large-scale trials suggest that AI companions may reduce symptoms of depression and anxiety while enhancing well-being^3,5^. Yet fundamental questions remain regarding the durability of these effects, the populations for whom they are most beneficial, and their comparability to established human-delivered treatments.

Understanding for whom and under what conditions AI-based interventions are most effective now represents a central challenge for clinical research. Decades of psychological research demonstrate that individual differences in interpersonal orientation and social context strongly influence how people engage with and benefit from support^6^. These dynamics may be even more salient in digital contexts, where relational conditions differ fundamentally from human-delivered care. Unlike therapists or peers, AI companions provide judgment-free, always-available interactions that unfold at the user’s chosen pace. Such distinctive features raise the possibility that specific relational profiles align more closely with the affordances of AI, potentially moderating clinical outcomes.

Attachment theory offers a particularly useful framework for examining these dynamics. Bowlby proposed that early caregiving experiences give rise to internal working models of relationships that shape expectations of availability, responsiveness, and support^7^. These orientations influence how individuals regulate emotions and seek comfort across the lifespan. Securely attached individuals generally approach relationships with trust and confidence, turning to others for support when distressed. Insecurely attached individuals, by contrast, may experience difficulties in forming or sustaining supportive connections.

Extensive empirical research demonstrates that secure attachment is associated with healthier relationship outcomes, including greater intimacy, trust, and relationship stability, as well as better life outcomes more broadly, such as effective emotion regulation, adaptive coping, and resilience in the face of stress. In contrast, insecure attachment patterns (anxious or avoidant) reliably predict difficulties in close relationships, heightened sensitivity to stress, less effective emotion regulation, and increased vulnerability to anxiety, depression, and other forms of psychological distress^6,8^.

Those with anxious attachment often display heightened concerns about rejection and abandonment, accompanied by an amplified need for reassurance. Because AI companions provide consistent availability and responsiveness without the risk of burdening others, they may serve as a particularly suitable support channel for this group^9^. Avoidantly attached individuals, who characteristically value autonomy and emotional distance, often resist dependence on human relationships. AI companions, which allow users to engage in supportive dialogue while retaining control and minimizing relational demands, may align well with avoidant preferences. In contrast, securely attached individuals may benefit more from human-delivered interventions that offer authentic mutual attunement and shared experience.

Recent findings support this view: anxious attachment predicts stronger intentions to use conversational agents, while avoidant attachment is linked to more distant patterns of interaction^10,11^. Anxious users may also form dependent emotional bonds with AI companions, whereas secure attachment is associated with more balanced use^12^. These results suggest that relational orientations guiding human interactions similarly shape engagement with AI-based interventions.

Beyond attachment, relational context also shapes mental health and intervention response. Loneliness, defined as the perceived lack of meaningful social connection, is a strong predictor of depression, anxiety, and poor well-being^13^. It is linked to heightened stress reactivity, impaired emotion regulation, and greater vulnerability to mental illness^14^. AI companions may provide compensatory connection through daily engagement, emotional presence, and continuity. Lonely individuals often attribute social presence to digital agents and find these interactions emotionally meaningful despite their artificiality^15^. Thus, they may experience disproportionate benefits from AI support. At the same time, reliance on AI could discourage cultivating human relationships and risk reinforcing isolation^16^. More research is needed to clarify whether loneliness enhances or complicates AI’s impact.

Perceived social support, defined as the belief that one has reliable, caring others to rely upon, is another powerful relational determinant of mental health. High levels of perceived support are consistently associated with resilience, reduced psychological distress, and improved recovery from adversity^17^. Conversely, individuals who perceive low support are at heightened risk of depression, anxiety, and poorer overall functioning^13^. AI interventions may be particularly impactful for those with low perceived support by providing accessible, judgment-free interaction in contexts where human support is limited or absent.

Despite their theoretical importance, relational factors have rarely been tested as moderators of AI-based mental health interventions. This study focused on university students with clinically relevant symptoms, evaluating both the effectiveness of an AI companion and the moderating role of attachment, loneliness, and social support. Using a randomized controlled design, participants were assigned to an AI intervention, an integrative group therapy program, or a no-intervention control. Group therapy served not only as an active control but also as a relational comparator, offering authentic human connection and shared experience, in contrast to AI’s continuous accessibility, anonymity, and user-directed pacing. By comparing outcomes across these contexts, the study examined whether specific relational profiles align more closely with one form of support than another.

Beyond serving as a distinct relational environment, conversational AI may offer several advantages that are difficult to replicate in traditional weekly interventions. Unlike scheduled therapy sessions, AI support can be accessed at the moment distress occurs, allows for repeated interactions between sessions, and can adapt continuously to fluctuations in users’ emotional states. Emerging digital mental health research suggests that accessibility, frequency of engagement, and timely intervention may represent important mechanisms through which technology-based interventions exert their effects^5^. These characteristics may be particularly relevant during periods of ongoing stress, when support needs arise unpredictably and often outside formal treatment settings.

Participants in the digital intervention engaged with Kai, a conversational AI platform that integrates cognitive-behavioral therapy, acceptance and commitment therapy, dialectical behavior therapy, mindfulness-based stress reduction, positive psychology, and psychoeducation. In addition to structured protocols, the AI platform offers an open channel for supportive dialogue, allowing free-form conversations alongside guided exercises. This blend of protocol-driven content and user-directed interaction is designed to combine therapeutic structure with the accessibility and warmth of supportive engagement while leveraging AI’s scalability. Delivered through adaptive dialogue, daily mood check-ins, and interactive exercises, the AI-based intervention provides flexible, always-available support.

To ensure safe use, the conversational AI platform incorporates continuous risk monitoring supported by a human-in-the-loop escalation system: language-based algorithms flag potential indicators of acute distress, which are then reviewed by licensed clinicians who can initiate appropriate safety responses when necessary. Prior longitudinal studies have linked its use to significant improvements in well-being^18,19^. Building on this evidence, the present trial evaluated the effectiveness of this AI-based conversational intervention and the moderating role of attachment, loneliness, and perceived social support, compared with group therapy and a no-intervention control.

First, the AI-based conversational intervention was expected to improve psychological outcomes, including reductions in generalized anxiety and depressive symptoms and gains in well-being, relative to both the control condition and group therapy. This expectation was based on the intervention’s continuous availability, personalization, adaptive feedback, and capacity to provide support in real-world contexts as emotional needs arise. Second, these effects would be more pronounced among individuals with relational vulnerabilities, including high loneliness, low perceived social support, and insecure attachment, who may particularly benefit from the accessible and responsive nature of conversational AI.

By integrating clinical outcome measures with assessments of attachment, loneliness, and support, the study aimed to clarify both the overall effectiveness of AI-based care and the relational contexts in which it is most beneficial.

## Results

### Participants

The study focused on university students in Israel who reported emotional distress and expressed interest in receiving psychological support. This population was selected to represent young adults facing elevated levels of stress, mood difficulties, and relational strain, yet with no access to formal mental health services. Of the 1032 individuals assessed for eligibility, 55 were excluded: 17 did not meet inclusion criteria (5 were outside the eligible age range, and 12 scored below the predefined psychological distress threshold on the PHQ-4 screening measure), 5 declined participation, and 33 met exclusion criteria such as severe psychiatric disorder or concurrent psychotherapy.

The remaining 977 participants (mean age = 23.1 years, SD = 2.4, range = 18–32; 50.9% female) were randomized to one of three conditions: AI-based digital intervention (*n* = 329), face-to-face group therapy (*n* = 324), or waitlist control (*n* = 324). At the 3-month follow-up, 629 participants (64.4%) completed assessments. Participant flow throughout the trial is presented in Supplementary Fig. 1.

At baseline, participants reported elevated psychological distress relative to population norms, with mean scores of 7.90 (SD = 5.16) on the PHQ-9^20^ for depression, 7.39 (SD = 4.86) on the GAD-7^21^ for generalized anxiety, and 4.40 (SD = 4.25) on the short-form PCL-5^22^ for PTSD. These values indicate elevated psychological distress relative to nonclinical young-adult norms. Population reference samples in this age range typically report PHQ-9 means of ~2.5–3.5 (SD ≈ 4.0)^23^, GAD-7 means of ~3–5 (SD ≈ 4.5)^24^, and 4-item PCL-5 means around 4.0 (SD ≈ 4.1)^22^. Thus, the study sample’s PHQ-9 and GAD-7 scores are approximately one standard deviation above normative values, and their short-form PCL-5 score is slightly elevated relative to expectations for nonclinical young adults. Although the average severity falls within the “mild” range, participants were substantially more distressed than typical age-matched populations, consistent with the study’s inclusion criterion of self-reported emotional or psychological distress. No significant differences were found between conditions in demographic or mental health characteristics (all *p*s > 0.07). Baseline characteristics are summarized in Table 1.

**Table 1:** Baseline characteristics

	AI Intervention	Group therapy	Control	Statistic	*p*
	(*n* = 329)	(*n* = 324)	(*n* = 324)		
	Mean (*SD*)				
Gender					
Males, *n* (%)	147 (44.7%)	172 (53.1%)	161 (49.7%)	*χ*² = 4.68	0.10
Age (years)	22.88 (2.14)	23.15 (2.59)	23.25 (2.50)	*F* = 2.06	0.13
Socioeconomic status (SES)					
High SES *n* (%)	80 (24.3%)	59 (18.2%)	57 (17.6%)	*χ*² = 7.76	0.10
Middle SES *n* (%)	202 (61.4%)	201 (62%)	207 (63.9%)		
Low SES *n* (%)	47 (14.3%)	64 (19.8%)	60 (18.5%)		
Religion				*χ*² = 0.56	0.97
Jewish, *n* (%)	322 (97.9%)	316 (97.5%)	317 (97.8%)		
Muslim, *n* (%)	5 (1.5%)	6 (1.9%)	5 (1.5%)		
Christian, *n* (%)	1 (0.3%)	2 (0.6%)	2 (0.6%)		
Marital status				*χ*² = 2.93	0.82
Married *n* (%)	7 (2.1%)	6 (1.9%)	5 (1.5%)		
Single *n* (%)	203 (61.9%)	206 (63.6%)	211 (65.3%)		
Divorced *n* (%)	1 (0.3%)	2 (0.6%)	0 (0%)		
In Relationship (not married) *n* (%)	117 (35.7%)	110 (34%)	107 (33.1%)		
Generalized anxiety	7.22 (4.65)	7.47 (4.89)	7.50 (5.05)	*F* = 0.31	0.73
Depression	7.64 (4.78)	8.15 (5.30)	7.91 (5.40)	*F* = 0.80	0.45
PTSD	4.25 (3.69)	4.39 (4.55)	4.57 (4.47)	*F* = 0.45	0.64
Life Satisfaction	27.95 (4.39)	27.81 (4.88)	27.78 (4.53)	*F* = 0.13	0.88
Well-being	54.86 (16.60)	52.39 (13.15)	52.95 (12.54)	*F* = 2.71	0.07

*N* = 977

### Changes in mental health and well-being across conditions

From baseline to post-intervention (T1–T2), a repeated-measures MANCOVA controlling for age and gender revealed a significant condition × time interaction, Wilks’ *λ* = 0.82, *F*(10, 1936) = 20.66, *p* < .001, *η*² = 0.10. No interaction with age or gender was observed (Table 2). Univariate analyses with Bonferroni correction (*α* = 0.01) identified generalized anxiety as the outcome showing the largest intervention effect, *F*(2, 972) = 67.92, *p* < 0.001, *η*² = 0.12. Participants in the AI-based intervention reported greater reductions than both the group therapy (mean difference = –2.10, 95% CI [–2.59, –1.60], *p* < .001) and control (–2.08, 95% CI [–2.57, –1.58], *p* < .001) groups, with no difference between the latter two (*p* = 1.00).

**Table 2:** Means, standard deviations, and effect sizes for outcome measures by intervention group

Outcome	Group	T1 M (SD)	T2 M (SD)	T3 M (SD)	*F* (Time × Condition, T1-T2)	*p*	*η*²	*Cohen’s d T1-T2*	*Cohen’s d T1-T3*
**Generalized anxiety**	AI intervention	7.22 (4.65)	6.31 (4.41)	6.18 (4.29)	67.92	<0.001	0.12	−0.20	−0.23
	Group therapy	7.47 (4.89)	8.65 (5.19)	8.53 (4.64)				0.23	0.22
	Control	7.50 (5.05)	8.66 (5.31)	8.78 (5.13)				0.22	0.25
**Depression**	AI intervention	7.64 (4.78)	6.72 (5.15)	6.52 (5.11)	25.42	<0.001	0.05	−0.19	−0.23
	Group therapy	8.15 (5.30)	7.88 (5.33)	8.04 (5.09)				−0.05	−0.02
	Control	7.91 (5.40)	8.90 (5.61)	8.75 (5.00)				0.18	0.16
**PTSD**	AI intervention	4.25 (3.69)	3.51 (3.59)	3.41 (3.08)	0.38	0.686	0.001	−0.20	−0.25
	Group therapy	4.39 (4.55)	3.81 (4.20)	3.67 (3.43)				−0.13	−0.18
	Control	4.57 (4.47)	4.04 (4.29)	3.99 (3.34)				−0.12	−0.15
**Life Satisfaction**	AI intervention	27.95 (4.39)	28.98 (4.23)	28.71 (5.71)	20.01	<0.001	0.04	0.24	0.15
	Group therapy	27.81 (4.88)	27.83 (4.50)	26.73 (5.78)				0.004	−0.20
	Control	27.78 (4.53)	26.31 (4.36)	26.09 (6.00)				−0.33	−0.32
**Well-being**	AI intervention	54.86 (16.60)	61.40 (17.57)	60.99 (19.11)	25.87	<0.001	0.05	0.38	0.34
	Group therapy	52.39 (13.15)	53.10 (12.41)	52.95 (15.32)				0.06	0.04
	Control	52.95 (12.54)	50.39 (12.83)	49.31 (15.98)				−0.20	−0.25

Sample sizes at T1 and T2: AI intervention *n* = 329; group therapy *n* = 324; control *n* = 324. Sample sizes at T3: AI intervention *n* = 213*;* group therapy *n* = 210*;* control *n* = 206.

Depression followed a similar pattern, *F*(2, 972) = 25.42, *p* < .001, *η*² = .05: the AI-based intervention outperformed control (–1.91, 95% CI [–2.55, –1.26], *p* < 0.001), and group therapy also exceeded control (–1.25, 95% CI [–1.90, –0.61], *p* < 0.001). The comparison between the AI-based and group therapy conditions was weaker (–0.65, 95% CI [–1.30, –0.01], *p* = 0.046) and did not reach significance under the stricter Bonferroni threshold (*α* = 0.01). PTSD symptoms showed no evidence of differential change, *F*(2, 972) = 0.38, *p* = 0.686, *η*² = 0.001.

Positive functioning outcomes displayed consistent condition effects. Well-being improved significantly, *F*(2, 972) = 25.87, *p* < 0.001, *η²* = 0.05. Participants in the AI-based intervention reported larger gains than both group therapy (+5.63, 95% CI [2.57, 8.69], *p* < 0.001) and control (+8.92, 95% CI [5.86, 11.98], *p* < 0.001), with group therapy also surpassing control (+3.28, 95% CI [0.21, 6.36], *p* = 0.031). Life satisfaction showed a similar pattern, *F*(2, 972) = 20.01, *p* < 0.001, *η*² = 0.04, with greater improvements for the AI-based intervention compared to the control group (+2.50, 95% CI [1.53, 3.46], *p* < 0.001) and group therapy (+1.01, 95% CI [0.05, 1.98], *p* = 0.036). Group therapy also showed greater improvement than the control group (+1.49, 95% CI [0.52, 2.46], *p* < 0.001). Within-group effect sizes indicated small improvements in the AI-based intervention group for generalized anxiety (*d* = –0.20), depression (*d* = –0.19), and PTSD (*d* = –0.20), with larger gains for life satisfaction (*d* = 0.24) and well-being (*d* = 0.38).

**Fig. 1:**
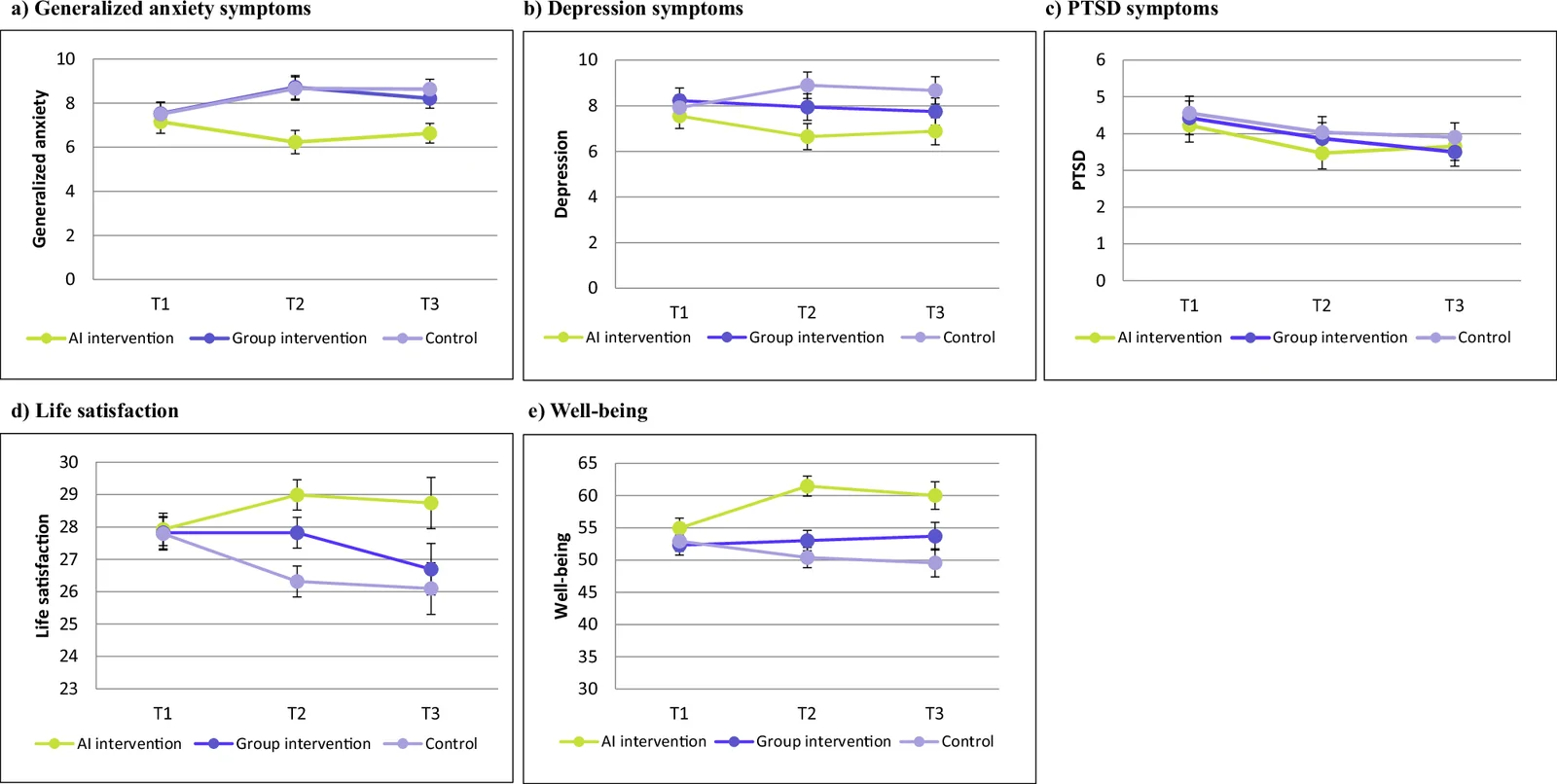
Changes in mental health and well-being outcomes across the AI intervention, group therapy, and control conditions. Mean scores and 95% confidence intervals are shown for **a** generalized anxiety symptoms (GAD-7), **b** depression symptoms (PHQ-9), **c** PTSD symptoms (PCL-5 short form), **d** life satisfaction (BMSLSS), and **e** well-being (WHO-5) at baseline (T1), post-intervention (T2), and 3-month follow-up (T3). Significant condition-by-time interactions were observed for generalized anxiety symptoms, depression symptoms, life satisfaction, and well-being, whereas no significant interaction was observed for PTSD symptoms. Estimated marginal means were adjusted for age and gender. Sample sizes were AI intervention (*n* = 329 at T1/T2; *n* = 213 at T3), group therapy (*n* = 324 at T1/T2; *n* = 210 at T3), and control (*n* = 324 at T1/T2; *n* = 206 at T3).

At the 3-month follow-up (T3), a one-way MANCOVA controlling for baseline measures, age, and gender revealed a significant overall condition effect, Wilks’ *λ* = 0.82, *F*(10, 1230) = 12.94, *p* < 0.001, *η*² = 0.09, with neither age nor gender reaching significance. Changes in outcome trajectories across the three conditions are presented in Fig. 1.

Univariate models indicated that intervention differences persisted. For generalized anxiety, there was a significant condition effect, *F*(2, 619) = 21.33, *p* < 0.001, *η*² = 0.06. Participants in the AI-based intervention reported significantly lower symptoms compared to both group therapy (mean difference = –1.58, 95% CI [–2.36, –0.80], *p* < 0.001) and the control group (–2.01, 95% CI [–2.78, –1.23], *p* < 0.001), while the latter two groups did not significantly differ (*p* = 0.56).

Depression also showed a significant condition effect, *F*(2, 619) = 8.50, *p* < 0.001, *η*² = 0.03. Scores were lower in the AI-based intervention compared to the control group (–1.79, 95% CI [–2.83, –0.75], *p* < 0.001), but the comparison with group therapy (–0.86, 95% CI [–1.90, 0.18], *p* = 0.14) and the contrast between group therapy and control (–0.93, 95% CI [–1.97, 0.11], *p* = 0.10) were not significant. PTSD symptoms did not differ across conditions, *F*(2, 619) = 1.05, *p* = 0.35, *η*² = 0.003.

Positive functioning outcomes demonstrated sustained benefits. Life satisfaction differed significantly between groups, *F*(2, 619) = 11.56, *p* < 0.001, *η*² = 0.04. Participants in the AI-based intervention reported greater improvements than both group therapy (mean difference = 2.05, 95% CI [0.66, 3.43], *p* = 0.001) and the control group (2.64, 95% CI [1.26, 4.03], *p* < 0.001), whereas group therapy and control did not differ (*p* = 0.90). Well-being showed the strongest pattern, *F*(2, 619) = 22.80, *p* < 0.001, *η*² = 0.07. Participants in the AI-based intervention reported higher scores compared to both group therapy (6.31, 95% CI [2.56, 10.06], *p* < 0.001) and control (10.46, 95% CI [6.71, 14.20], *p* < 0.001), and group therapy also exceeded control (4.15, 95% CI [0.41, 7.88], *p* = 0.024).

### Clinical significance and proportion below clinical thresholds

To extend these findings, we examined the clinical significance of changes across conditions using established clinical cutoffs (≥10) for the PHQ-9, GAD-7, and the short-form PCL-5 (Table 3). Consistent with the symptom-level analyses, the AI-based intervention produced the highest proportions of participants who shifted from the clinical to the non-clinical range at T2. Among those who met the clinical threshold at baseline, 46.5% moved below the cutoff for depression, 56.5% for generalized anxiety, and 58.3% for PTSD, exceeding rates in both comparison conditions for depression and generalized anxiety. Group therapy showed moderate proportions moving below the clinical threshold for depression (26.4%) and generalized anxiety (10.4%), while shifts in PTSD status were similar across conditions. The control condition demonstrated the lowest proportions of participants moving below the clinical cutoff for depression (14.1%) and generalized anxiety (7.3%). These differences were statistically significant for depression and generalized anxiety, with large between-group effects at post-intervention (overall *χ*² = 29.46 for depression; *χ*² = 81.08 for anxiety, both *p* < 0.001).

**Table 3:** Proportion of participants shifting from clinical (≥10) to non-clinical range (<10)

Measure	Condition	Baseline clinical *n* (%)	Post-intervention non-clinical *n* (%)	*χ*² vs AI (*p*)	Follow-up non-clinical *n* (%)	*χ*² vs AI (*p*)
**Depression (PHQ-9)**	AI	99 (30.1%)	46 (46.5%)	—	29/57 (50.9%)	—
	Group therapy	110 (34.0%)	29 (26.4%)	*χ*² = 9.15, *p* = 0.003	28/73 (38.4%)	*χ*² = 2.04, *p* = 0.15
	Control	128 (39.5%)	18 (14.1%)	*χ*² = 28.95, *p* < 0.001	31/83 (37.3%)	*χ*² = 2.53, *p* = 0.11
	Overall *χ*²	—	—	*χ*² = 29.46, *p* < 0.001	—	*χ*² = 2.95, *p* = 0.23
**Generalized anxiety (GAD-7)**	AI	92 (28.0%)	52 (56.5%)	—	27/53 (50.9%)	—
	Group therapy	96 (29.6%)	10 (10.4%)	*χ*² = 45.18, *p* < 0.001	14/68 (20.6%)	*χ*² = 12.25, *p* < 0.001
	Control	110 (34.0%)	8 (7.3%)	*χ*² = 58.20, *p* < 0.001	10/69 (14.5%)	*χ*² = 18.85, *p* < 0.001
	Overall *χ*²	—	—	*χ*² = 81.08, *p* < 0.001	—	*χ*² = 22.39, *p* < 0.001
**PTSD (PCL-5 Short)**	AI	36 (10.9%)	21 (58.3%)	—	9/16 (56.2%)	—
	Group therapy	47 (14.5%)	25 (53.2%)	*χ*² = 0.22, *p* = 0.64	23/32 (71.9%)	*χ*² = 1.17, *p* = 0.28
	Control	46 (14.2%)	25 (54.3%)	*χ*² = 0.13, *p* = 0.72	23/31 (74.2%)	*χ*² = 1.56, *p* = 0.21
	Overall *χ*²	—	—	*χ*² = 0.23, *p* = 0.89	—	*χ*² = 1.74, *p* = 0.42

T1 and T2 sample sizes were AI intervention *n* = 329, group therapy *n* = 324, and control *n* = 324. Follow-up sample sizes vary due to differential attrition across conditions.

At follow-up, the AI-based intervention continued to show the highest proportions of participants below the clinical threshold for depression (50.9%) and generalized anxiety (50.9%), with a significant between-group difference only for generalized anxiety. Differences across conditions for depression and PTSD at follow-up were not statistically significant. Follow-up comparisons were based on varying subgroup sample sizes due to attrition.

### Adherence and satisfaction

Engagement with the AI-based intervention was high throughout the study period. On average, participants sent 221 messages (SD = 148.9) and interacted with the platform on an average of 3.0 days per week. Overall, 43% engaged on four or more days, 30% on three days, 21% on two days, and 6% on one day weekly. Retention analyses showed a gradual decline in active use across the 12-week intervention, from 100% at week 1 to 83% at week 4, 72% at week 8, and 61% at week 12.

**Fig. 2:**
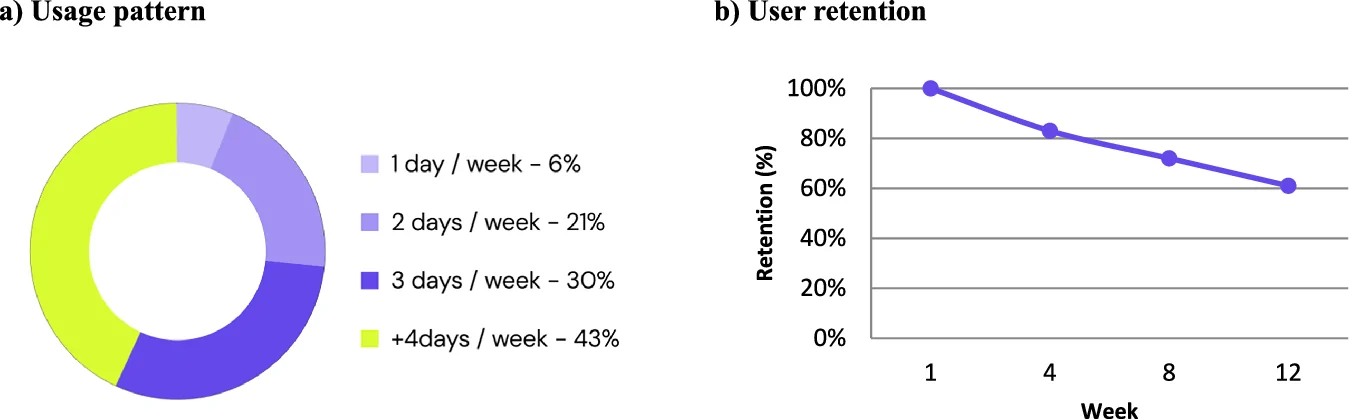
Patterns of user engagement and retention within the AI conversational platform. **a** Weekly usage frequency across the intervention period, showing the proportion of participants who interacted with the system on one, two, three, or four or more days per week. **b** Retention curve showing the proportion of participants who remained active on the platform at weeks 1, 4, 8, and 12. Engagement was high overall, with most participants using the platform on three or more days weekly and 61% remaining active through the full 12-week intervention period.

Participants wrote an average of 1642.84 words per week (SD = 1348.1), with weekly totals ranging from 5 to 5227 words. Time-use estimates indicated that participants spent an average of 82.14 min per week interacting with the AI platform (SD = 67.41), equivalent to approximately 1.37 h. Fig. 2 shows the cumulative percentage of participants who reached different engagement thresholds.

Participant feedback was highly positive: 91.8% reported feeling connected to the material, 90.6% indicated learning new coping strategies, and 75.7% expressed a desire to continue using the AI system after the trial. In addition, 92.4% described the interactions as supportive and non-judgmental, emphasizing the platform’s accessibility and ease of use in daily life.

In the group therapy condition, adherence and satisfaction were also high. Attendance records indicated that participants attended an average of 10.3 out of 12 sessions, with 233 participants (71.9%) completing at least 10 sessions. Participant satisfaction was similarly strong: 91.1% reported that the sessions were helpful, 87.7% endorsed learning new coping strategies, and 58.4% expressed interest in continuing group sessions in the future. Qualitative feedback highlighted the value of peer sharing and social connection, with participants noting that being part of a supportive group reduced feelings of isolation and normalized their struggles. At the same time, some participants described challenges related to time demands and commuting to fixed weekly meetings as barriers to full engagement.

### Moderation effects of relational factors

Building on the primary intervention effects, we next examined whether relational psychological factors shaped participants’ responses to the interventions. Moderation analyses were conducted using the PROCESS macro for SPSS (*Model 1*)^25^, testing whether baseline levels of loneliness, perceived social support, and attachment styles moderated the effects of the interventions on mental health and well-being. Standardized regression coefficients for all models are presented in Supplementary Table 1.

The AI-based intervention showed consistent main effects across outcomes. It was negatively associated with generalized anxiety (*β* = –0.33 to –0.34, *p* < 0.001) and depression (*β* = –0.17, *p* < 0.001), and positively associated with well-being (*β* = 0.20 to 0.21, *p* < 0.001) and life satisfaction (*β* = 0.15 to 0.16, *p* < 0.001). No significant main effects were observed for PTSD symptoms (*β* = –0.02, *p* > 0.05).

**Fig. 3:**
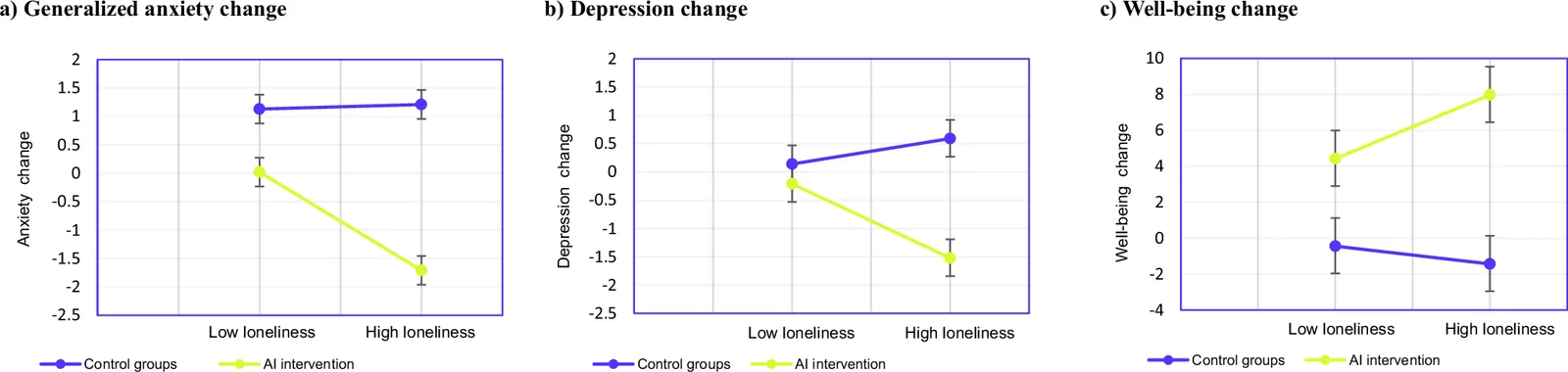
Moderation effects of loneliness. Interaction plots display mean change scores with ninety five percent confidence intervals from baseline to post-intervention in generalized anxiety (GAD-7), depression (PHQ-9), and well-being (WHO-5) across low and high loneliness (UCLA Loneliness Scale short form) for the AI intervention and Control groups. **a** Mean change in anxiety across loneliness levels. Significant loneliness-by-condition interaction effects were observed, such that generalized anxiety reductions were larger in the AI intervention at higher loneliness. **b** Mean change in depression across loneliness levels. Significant moderation effects were detected, with greater symptom reduction in the AI intervention among participants reporting higher loneliness. **c** Mean change in well-being across loneliness levels. Significant loneliness-by-condition interaction effects indicated larger improvements in the AI intervention at higher loneliness.

Loneliness significantly moderated intervention effects on generalized anxiety (*β* = –0.15, *p* < 0.001), depression (*β* = –0.12, *p* < 0.001), and well-being (*β* = 0.06, *p* = 0.04) (Fig. 3). For anxiety, participants in the AI-based intervention experienced larger reductions at high levels of loneliness (*B* = –2.92, SE = 0.24, *t* = –12.05, *p* < 0.001, 95% CI [–3.39, –2.44]) than at low levels (*B* = –1.14, SE = 0.25, *t* = –4.44, *p* < 0.001, 95% CI [–1.61, –0.62]). For depression, effects strengthened from *B* = –1.23 (SE = 0.23, *t* = –5.29, *p* < 0.001, 95% CI [–1.69, –0.78]) at low loneliness to *B* = –2.11 (SE = 0.24, *t* = –8.70, *p* < 0.001, 95% CI [–2.57, –1.65]) at high loneliness. For well-being, gains increased from *B* = 4.88 (SE = 1.58, *t* = 3.09, *p* = 0.002, 95% CI [1.78, 7.97]) at low loneliness to *B* = 9.42 (SE = 1.52, *t* = 6.19, *p* < 0.001, 95% CI [6.44, 12.41]) at high loneliness.

Perceived social support moderated only generalized anxiety outcomes (*β* = 0.10, *p* = 0.02). The AI-based intervention produced stronger anxiety reductions among participants with lower support (*B* = –2.65, SE = 0.30, *t* = –8.59, *p* < 0.001, 95% CI [–3.26, –2.06]) than among those with higher support (*B* = –2.11, SE = 0.31, *t* = –6.91, *p* < 0.001, 95% CI [–2.74, –1.49]), suggesting that the intervention may buffer against insufficient social resources.

**Fig. 4:**
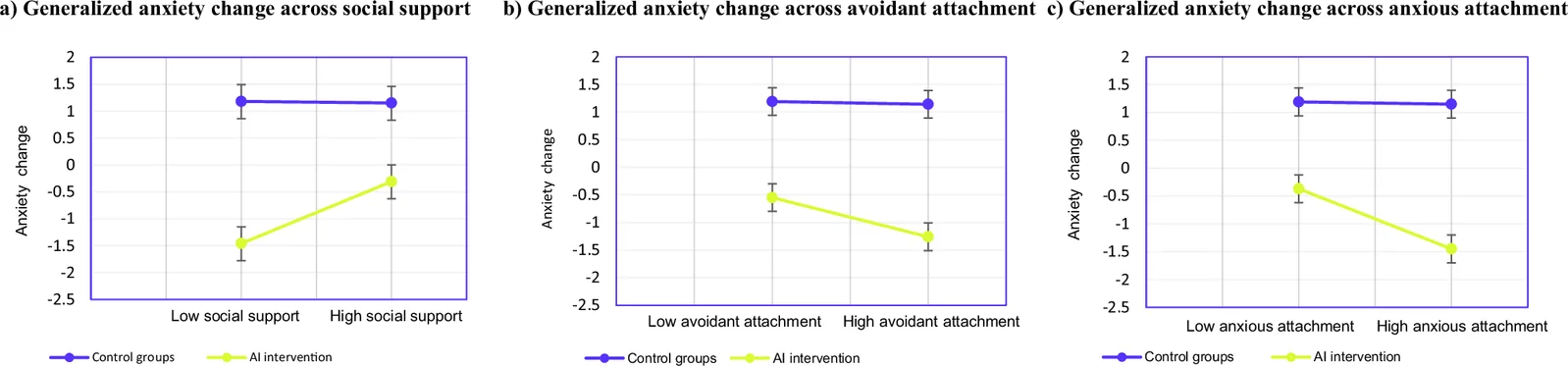
Moderation effects of social support and attachment styles on anxiety change. Interaction plots display mean change scores with ninety five percent confidence intervals from baseline to post-intervention in anxiety (GAD-7) across levels of perceived social support (MSPSS), avoidant attachment (ECR-R Avoidance), and anxious attachment (ECR-R Anxiety) for the AI intervention, Group therapy, and Control conditions. **a** Mean change in anxiety across levels of social support. Significant support-by-condition interaction effects indicated larger anxiety reductions in the AI intervention among participants reporting lower social support. **b** Mean change in anxiety across levels of avoidant attachment. Significant moderation effects were observed, such that anxiety reductions in the AI intervention were greater among participants with higher avoidant attachment. **c** Mean change in anxiety across levels of anxious attachment. Significant anxious-attachment-by-condition interaction effects showed larger anxiety reductions in the AI intervention among participants reporting higher anxious attachment.

Attachment styles also moderated generalized anxiety outcomes. For avoidant attachment (*β* = –0.07, *p* = 0.04), reductions were greater at high avoidance (*B* = –2.41, SE = 0.29, *t* = –8.60, *p* < 0.001, 95% CI [–2.90, –1.91]) than at low avoidance (*B* = –1.99, SE = 0.29, *t* = –6.83, *p* < 0.001, 95% CI [–2.26, –1.26]). For anxious attachment (*β* = –0.09, *p* = 0.004), reductions were likewise stronger at high levels (*B* = –2.60, SE = 0.25, *t* = –10.30, *p* < 0.001, 95% CI [–3.10, –2.11]) than at low levels (*B* = –1.55, SE = 0.26, *t* = –6.18, *p* < 0.001, 95% CI [–2.06, –1.06]) (Fig. 4).

Taken together, moderation effects were most pronounced for generalized anxiety, with all four relational factors (loneliness, social support, avoidant attachment, and anxious attachment) significantly influencing intervention outcomes. Loneliness also moderated effects on depression and well-being, whereas no moderation was observed for PTSD or life satisfaction. Overall, these findings suggest that the AI-based intervention yielded its strongest benefits among individuals with relational vulnerabilities, particularly those experiencing high loneliness, limited social support, and elevated attachment insecurity.

To further contextualize these moderation patterns, we examined whether engagement with the AI system differed between low- and high-loneliness subgroups. Participants were classified into low (*z* ≤ –1; *n* = 71) and high (*z* ≥ +1; *n* = 51) loneliness groups based on standardized loneliness scores. Participants with low loneliness generated an average of 1216.9 words per week (SD = 1117.1), whereas those with high loneliness produced 2489.90 words (SD = 1642.01), a significant difference, *t*(120) = 5.10, *p* < 0.001. Estimated weekly time spent interacting with the AI system showed a similar pattern: the low loneliness group spent 60.84 min per week interacting with the AI platform (SD = 55.86), compared with 124.49 min in the high loneliness group (SD = 82.10; *p* < 0.001). Together, these findings indicate markedly greater engagement among participants with higher loneliness.

Exploratory moderation analyses indicated that relational factors also shaped responses in the group therapy condition (see Supplementary Table 2). Loneliness significantly moderated generalized anxiety outcomes (*β* = –0.11, *p* < 0.001), such that participants with higher loneliness experienced greater reductions in anxiety symptoms than those with lower loneliness. A weaker moderation was observed for depression (*β* = –0.07, *p* = 0.038), showing a similar though less pronounced pattern.

For well-being, both loneliness (*β* = –0.08, *p* = 0.009) and perceived social support (*β* = –0.11, *p* = 0.013) significantly moderated intervention effects. Conditional effect analyses revealed that group therapy improved well-being across participants, but the magnitude of improvement was smaller among those with high loneliness or low perceived social support. In other words, while lonely or socially unsupported participants still benefited from the group intervention, their gains in well-being were less pronounced compared with peers reporting lower loneliness and stronger social networks. No significant moderation was observed for attachment styles.

### Engagement as a potential mediator

To better understand how relational vulnerabilities may have shaped outcomes, we conducted an exploratory mediation analysis examining whether participant engagement with the AI-based intervention mediated the association between relational risk factors and reductions in generalized anxiety symptoms. A structural equation model was specified in which loneliness, low social support, and anxious and avoidant attachment loaded onto a latent relational factor, which subsequently predicted engagement with the AI-based intervention (operationalized as the total number of messages sent; Supplementary Fig. 2).

The relational factor was positively associated with engagement (*β* = 0.41, *p* < 0.001), and engagement, in turn, significantly predicted reductions in generalized anxiety (*β* = –0.44, *p* < 0.001). When engagement was included in the model, the direct path from the relational factor to anxiety change was no longer significant (*c*′ path: *β* = –0.02), whereas the total effect remained significant (*c* path: *r* = –0.22, *p* = 0.003), consistent with full mediation. Model fit indices indicated acceptable fit: *χ*² = 18.85, *p* = 0.009, CFI = 0.94, TLI = 0.87, RMSEA = 0.072, SRMR = 0.093. Taken together, this exploratory model suggests that relational vulnerabilities were linked to greater reductions in generalized anxiety primarily through increased engagement with the AI-based intervention. Because this analysis was conducted post hoc and was not preregistered, these findings should be interpreted with caution but may indicate a plausible mechanism for future investigation.

### Relational vulnerability, engagement, and clinical outcomes

To clarify whether relational vulnerabilities predicted outcomes independently of engagement or primarily through increased use of the AI intervention, we conducted a series of hierarchical regressions predicting T2 generalized anxiety, depression, PTSD, life satisfaction, and well-being. In Step 1, models included baseline symptom severity together with loneliness, perceived social support, and anxious and avoidant attachment. In Step 2, we added overall engagement, operationalized as the total number of messages participants sent to the AI system across the 12-week intervention. Key models for generalized anxiety symptoms and depression are presented in Table 4, and the full set of models is provided in Supplementary Table 3.

**Table 4:** Predictors of generalized anxiety and depression outcomes at post-intervention

Generalized anxiety (T2)						Depression (T2)					
Predictor	B (SE)	*β*	*t*	*P* value	95% CI for B	**Predictor**	B (SE)	*β*	*t*	*P* value	95% CI for B
Constant	5.38 (1.82)		2.96	0.003	[1.803, 8.961]	Constant	4.91 (1.95)		2.51	0.012	[1.079, 8.746]
Baseline anxiety	0.55 (0.04)	0.58	12.43	<0.001	[0.464, 0.639]	Baseline depression	0.70 (0.05)	0.65	14.87	<0.001	[0.608, 0.794]
Loneliness	0.25 (.14)	0.11	1.75	0.08	[−0.031, 0.520]	Loneliness	0.17 (0.15)	0.06	1.09	0.28	[−0.135, 0.470]
Social support	−0.44 (0.27)	−0.09	−1.62	0.11	[−0.977, 0.095]	Social support	−0.53 (0.29)	−0.09	−1.80	0.07	[−1.099, 0.049]
Avoidant attachment	0.002 (.18)	0.001	0.014	0.99	[−0.356, 0.361]	Avoidant attachment	−0.05 (0.20)	−0.01	−0.27	0.79	[−0.437, 0.331]
Anxious attachment	−0.03 (0.18)	−0.007	−0.14	0.89	[−0.374, 0.323]	Anxious attachment	−0.03 (.19)	−0.006	−0.14	0.89	[−0.399, 0.345]
Engagement	−0.01 (0.001)	−0.39	−8.31	<0.001	[−0.014, −0.009]	Engagement	−0.009 (0.001)	−0.27	−6.25	<0.001	[−0.012, −0.006]

*B* unstandardized coefficient, *β* standardized coefficient, *CI* confidence interval.

Final-step hierarchical regression models among AI intervention participants (*n* = 329).

Across all outcomes, baseline symptom severity was the strongest predictor (*β*s = 0.54 to 0.70, all *ps* < 0.001). Once baseline symptom levels were controlled, relational factors showed minimal or nonsignificant direct effects (all *ps* > 0.07). Adding engagement significantly improved model fit for every outcome: generalized anxiety (Δ*R*² = 0.12, *p* < 0.001), depression (Δ*R*² = 0.06, *p* < 0.001), PTSD (Δ*R*² = 0.03, *p* < 0.001), life satisfaction (Δ*R*² = 0.006, *p* = 0.03), and well-being (Δ*R*² = 0.03, *p* < 0.001). Engagement emerged as a consistent independent predictor: sending more messages to the AI system was associated with greater reductions in generalized anxiety (*β* = –0.39, *p* < 0.001), depression (*β* = –0.27, *p* < 0.001), and PTSD (*β* = –0.17, *p* < 0.001), as well as greater increases in life satisfaction (*β* = 0.09, *p* = 0.03) and well-being (*β* = 0.20, *p* < 0.001).

Taken together, these findings indicate that relational vulnerabilities did not directly predict mental health outcomes once engagement was accounted for. Instead, their influence appears to operate indirectly through higher levels of sustained messaging engagement, consistent with the mediation pattern reported earlier, suggesting that individuals with greater relational risk may benefit more because they engage more intensively with the AI-based intervention.

## Discussion

This randomized controlled trial provides evidence that conversational AI can contribute meaningfully to the treatment of mental health difficulties among young adults. Both the AI-based conversational agent and group therapy were associated with improvements across several outcomes compared with the waitlist control. However, the pattern of effects differed across conditions.

The AI platform produced broader and more consistent benefits than the comparison conditions. It yielded significant improvements in generalized anxiety, depression, well-being, and life satisfaction relative to the control group, and outperformed group therapy on generalized anxiety, well-being, and life satisfaction at both post-intervention and follow-up. In contrast, group therapy showed more selective effects: it reduced depression relative to control at post-intervention and enhanced well-being and life satisfaction, with only the well-being gains persisting to follow-up. Neither intervention significantly affected PTSD symptoms.

Beyond average symptom reductions, the clinical significance analysis offers insight into the proportion of participants who achieved meaningful improvement. At post-intervention, 46.5% of participants in the AI condition who entered the study in the clinical range no longer met the clinical cutoff for depression, and 56.5% showed a comparable shift for generalized anxiety. These proportions were notably lower in group therapy (26.4% and 10.4%, respectively) and in the control condition (14.1% and 7.3%). For PTSD, the pattern differed: recovery rates were similar across conditions (58.3% in the AI arm and approximately 53–54% in the comparison groups). Taken together, these findings suggest that the AI-based intervention supported clinically meaningful improvement for a larger proportion of participants in the two most prevalent domains, depression and generalized anxiety.

The proportion of participants in the AI-based intervention who moved out of the clinical range provides a clear indicator of the magnitude of meaningful change achievable through scalable, low-barrier digital support. These estimates can help clinicians and service providers determine when AI tools may be appropriate as first-line or adjunctive options, particularly for individuals with elevated but non-complex presentations who may benefit from immediate, adaptive support. For health systems and policymakers, these rates offer concrete benchmarks for estimating the potential population-level impact of integrating AI-based interventions into stepped-care models, including their capacity to absorb early-stage demand and reduce pressure on oversubscribed human-delivered services.

This favorable psychological adaptation aligns with prior empirical findings showing that daily digital interventions, including cognitive behavioral therapy (CBT)^26^ and mindfulness-based practices^27^, can yield beneficial outcomes. Specifically, the AI-based intervention in this study employed an integrative framework that combined multiple evidence-based modalities^28,29^. Such multimodal approaches have been shown to produce more substantial and sustained improvements than single-method applications, particularly when integration is systematic and theoretically grounded.

One of the most consistent findings was the stronger impact of the AI-based intervention on generalized anxiety outcomes. Anxiety symptoms often fluctuate over short time frames, making timely support critical. The intervention’s 24/7 availability allowed participants to access coping tools during moments of acute stress. This design aligns with the concept of ecological momentary interventions, which deliver support in real time and within the flow of daily life^30^. Prior digital health research has highlighted this delivery mode as a key mechanism for symptom regulation. Group therapy, while beneficial in structured weekly sessions, could not provide such immediacy. The ability to deliver interventions precisely when needed may help explain the AI-based platform’s superior performance on anxiety outcomes across both time points.

The larger effects observed for well-being outcomes may reflect both intervention-specific and measurement-related factors. The AI-based intervention included several components that directly targeted positive psychological functioning, including reflective dialogue, meaning-making, gratitude, strengths use, and continuous emotional support. These processes may influence well-being and life satisfaction more directly than symptom-focused outcomes, which often reflect more persistent patterns of psychological distress. At the same time, improvements in well-being can also function as mechanisms of symptom change: prior work on multicomponent positive psychology interventions suggests that increases in positive emotions, meaning, optimism, and self-compassion can mediate reductions in anxiety and depression^31^. Thus, the stronger effects on well-being may represent both a proximal outcome of the intervention and a pathway through which later symptom improvement occurs. In addition, well-being measures may be more sensitive to early positive changes in daily functioning, perceived coping resources, and emotional support, whereas reductions in generalized anxiety or depressive symptoms may require more sustained or intensive change.

Personalization may also have contributed to the AI-based intervention’s relative advantage. The platform adjusted its content according to baseline assessments and continued to adapt based on ongoing user interactions. Prior work shows that tailoring digital interventions enhances engagement and clinical efficacy^32,33^. Group therapy, while effective in providing interpersonal support and structure, followed a semi-structured protocol designed to address shared needs within a fixed agenda, which may have limited opportunities for individualized focus. For some participants, particularly those with specific coping challenges, this adaptive and personalized approach may have contributed to the stronger effects observed in the AI-based intervention.

The relative strengths of the AI-based intervention may also reflect differences in perceived safety and stigma. Many young adults hesitate to engage in face-to-face services due to concerns about judgment or exposure^34^. Conversational AI can offer anonymity and nonjudgmental support, which may facilitate disclosure of sensitive experiences. Previous work has shown that individuals are often more willing to disclose stigmatized information to digital agents than to humans^35^. For some participants, this may have enhanced therapeutic engagement and depth of reflection in ways less accessible in peer-based formats such as group therapy.

Consistency and scalability are additional advantages observed in the AI-based intervention. In traditional group therapy, outcomes can vary depending on facilitator expertise, group cohesion, and participant attendance^36^. In contrast, the AI system was able to deliver content to many participants simultaneously, each receiving a personalized sequence of evidence-based tools within a unified therapeutic framework. Because its algorithmic delivery is not constrained by human availability, it provides consistent quality across users while remaining adaptive to individual needs. These qualities were reflected in the sustained engagement observed over time, suggesting that scalability and reliability may contribute to continued user participation. Such scalability is particularly valuable in contexts where demand for mental health support exceeds available professional capacity. For instance, recent research highlights that despite increasing need for counseling in university settings, many students are unable to access timely support^37^. Addressing this imbalance requires scalable and accessible interventions that can extend reach and complement existing human services.

The moderation findings also shed light on differential benefits across participants. Loneliness, attachment insecurity, and low perceived social support were not only associated with poorer baseline functioning but also amplified the positive effects of the AI-based intervention. Participants reporting higher loneliness, attachment anxiety, or attachment avoidance, as well as those perceiving lower social support, appeared to engage more intensively with the intervention and, in turn, experienced greater reductions in generalized anxiety. Interestingly, both anxiously and avoidantly attached individuals engaged more actively, differing from prior assumptions that avoidantly attached users would show lower trust or engagement with AI systems^38^. This discrepancy may reflect characteristics of the current sample, which included participants experiencing elevated symptoms and seeking emotional support, as well as the potential mitigating effect of the intervention’s anonymity on avoidant tendencies.

A consistent pattern emerged across outcomes: relational vulnerabilities such as loneliness, limited social support, and insecure attachment were not directly associated with symptom improvement. Rather, their relevance appeared primarily indirect, operating through heightened engagement with the AI-based intervention. Participants with greater relational risk sent substantially more messages over the 12-week program, and this increased engagement predicted reductions in generalized anxiety, depression, and PTSD, as well as gains in life satisfaction and well-being. Engagement accounted for additional variance in symptom change beyond baseline symptoms and relational characteristics, underscoring its role as a central mechanism linking relational vulnerabilities to improved outcomes. These findings align with evidence that the intensity of user interaction is a key driver of change in digital mental health interventions^39^ and highlight the importance of supporting sustained engagement when implementing AI-based interventions at scale.

At the same time, these findings raise important questions about the broader role of digital relational agents in the social lives of young adults. On the one hand, AI-based systems may serve as valuable tools for fostering a sense of safety, consistency, and responsiveness, qualities often lacking for individuals experiencing loneliness or relational insecurity. By offering empathic dialogue and adaptive support, such systems can encourage coping, self-reflection, and a sense of emotional stability. These functions mirror some of the benefits traditionally associated with human relationships and may be particularly helpful for individuals who face barriers to accessing social support offline.

On the other hand, excessive reliance on AI for emotional connection carries potential risks. Recent studies indicate that strong emotional attachment to conversational agents may, over time, exacerbate loneliness or reduce motivation to pursue real-world relationships^40,41^. Users with high attachment anxiety or a tendency to anthropomorphize AI by viewing it as capable of emotions or intentions may become overly dependent on digital agents, potentially reinforcing avoidance of human contact and deepening existing social challenges. Moreover, while the intervention simulated empathic dialogue, it cannot replace the reciprocity, unpredictability, and growth opportunities inherent in genuine human interactions.

These considerations highlight the importance of designing digital interventions that encourage engagement with real-world social contexts. In the present study, the AI system incorporated supportive prompts, gratitude reflections, and daily exercises aimed at enhancing awareness of relational resources beyond the platform. Such design features may help prevent social withdrawal by positioning AI not as a substitute for human connection but as a bridge toward it. Encouraging users to transfer skills acquired in digital interactions into face-to-face relationships is essential for promoting long-term well-being.

At the same time, the group therapy condition revealed complementary patterns of change, underscoring the distinct yet convergent pathways through which human and digital interventions can support mental health. Participants who reported higher loneliness showed greater reductions in generalized anxiety and, to a lesser extent, depression, indicating that the group format can provide meaningful relief even for those who initially feel socially isolated. The structured therapeutic exercises and coping strategies offered in the group context were effective for managing distress, while receiving concrete tools such as cognitive reframing or relaxation strategies, alongside peer validation, may have helped normalize experiences of anxiety and reduce depressive symptoms.

Group therapy also improved well-being across participants, but the magnitude of improvement was smaller among those with high loneliness or low perceived social support. One possible explanation is that group-based formats may be particularly effective for reducing acute distress, whereas fostering broader aspects of well-being, such as meaning, purpose, or long-term growth, may require more sustained engagement or individualized focus, especially for individuals who lack consistent social support in their daily lives. Taken together, these findings highlight both the potential and the boundaries of scalable digital and group-based interventions in addressing complex mental health needs.

Although the findings provide valuable insight into differential intervention effects, several limitations should be acknowledged when interpreting the results. All outcome measures were based on self-report, which may be influenced by expectancy effects, response biases, or shared method variance. Although retention at follow-up was high, variation in adherence and engagement across conditions may have influenced the magnitude of observed effects. Relatedly, the active conditions differed in treatment dose and delivery format: the AI-based intervention provided continuous, on-demand access and allowed repeated user-initiated engagement throughout the week, whereas the group intervention was delivered through scheduled weekly sessions. Thus, between-group differences may partly reflect differences in accessibility, immediacy, and opportunities for engagement, rather than intervention content alone. The AI-based intervention was also more individually tailored than the group intervention, as it adapted content based on onboarding data and ongoing conversational input, whereas group therapy followed a more structured format designed for shared delivery. This limits causal interpretation of between-group effects, as observed differences may partly reflect the degree of personalization rather than the therapeutic model itself. In addition, participants’ use of therapeutic techniques between group sessions was not systematically measured, limiting our ability to compare total treatment utilization across conditions. The 3-month follow-up limits conclusions about the durability and maintenance of outcomes over time.

The sample comprised young adults experiencing elevated distress but without acute psychiatric conditions, which constrains the generalizability of findings to more severe or clinically diverse populations. Group therapy was delivered in a brief, university-based format with limited session duration and intensity, and outcomes might differ in community or clinical settings where interventions are longer or more intensive. Importantly, PTSD symptoms did not improve in any condition, emphasizing the need for trauma-focused interventions that specifically target avoidance and re-experiencing processes^42^.

Finally, the study was conducted within the Israeli context, primarily among university students. Israel is characterized by chronic exposure to sociopolitical conflict, which may shape both baseline levels of distress and responsiveness to mental health interventions. Cultural norms surrounding emotional expression, help-seeking, and technology use may further influence engagement and treatment outcomes. Additionally, the study did not include objective behavioral or physiological measures of improvement, which could complement self-reported outcomes and provide a more comprehensive assessment of intervention effects. Future research should examine these interventions across more diverse cultural, linguistic, and geopolitical settings, incorporate multimodal outcome measures, and include longer follow-up periods to assess long-term effects and potential relapse prevention.

Taken together, the findings provide converging evidence that conversational AI can promote meaningful improvements in mental health and well-being among young adults experiencing elevated distress. The intervention’s capacity to deliver personalized, adaptive, and on-demand support highlights the growing potential of digital agents to supplement conventional therapy, especially in contexts of limited human resources. Importantly, the benefits extended beyond symptom reduction to include improvements in broader indicators of well-being and emotional functioning, suggesting that AI-based systems can engage mechanisms of self-reflection and coping similar to those observed in human-facilitated care.

At the same time, the complementary effects observed in group therapy underscore the enduring importance of human connection, empathy, and shared experience in psychological healing. The two modalities appear to address overlapping yet distinct mechanisms: conversational AI provides immediacy, personalization, and privacy, while group therapy offers relational depth, peer validation, and structured interpersonal feedback. These findings point toward the promise of integrative or stepped-care frameworks in which scalable AI-based interventions can serve as accessible entry points or adjuncts to human-delivered therapy. Such models may enhance efficiency and reach while preserving the relational and contextual richness of traditional care.

Beyond clinical implementation, the present results contribute to the emerging understanding of relational processes in human–AI interaction. The finding that individuals with higher loneliness or attachment insecurity engaged more deeply with the digital intervention suggests that psychological vulnerabilities may shape the perceived availability and responsiveness of AI agents. This observation aligns with attachment-based models of emotional regulation and extends them into the digital context, emphasizing the need to conceptualize digital mental health not only as technological innovation but also as a relational phenomenon.

Ethical and developmental considerations also arise from these findings. As conversational AI systems become increasingly sophisticated, there is a growing responsibility to ensure that such technologies support autonomy, social connection, and psychological growth rather than dependence or isolation. Integrating features that encourage users to translate digital coping skills into offline relationships, as implemented in the present design, may serve as one safeguard against potential risks. Continued interdisciplinary collaboration among clinicians, technologists, and ethicists will be essential to ensure that digital companions are developed and deployed in ways that enhance, rather than replace, the human foundations of mental health care.

Finally, future research should test these models across more diverse populations and cultural contexts, examine long-term trajectories of engagement and clinical outcomes, and incorporate objective behavioral and physiological markers to complement self-reported data. By advancing both mechanistic understanding and implementation science, the next generation of digital interventions can move beyond feasibility toward precision, ethics, and sustainability in mental health care delivery. Ultimately, the true promise of digital mental health lies not in replacing human connection but in extending its reach, bringing moments of care, empathy, and understanding to those who might otherwise remain unheard.

## Methods

### Study design and participants

The trial employed a three-arm randomized controlled design with equal allocation across conditions. Participants were 977 university students in Israel, recruited through social media campaigns, university mailing lists, and digital advertisements. Inclusion criteria were: (a) age 18–35 years; (b) current emotional or psychological distress, operationalized as a score of 3 or higher on either the anxiety or depression subscale of the Patient Health Questionnaire-4 (PHQ-4) administered during screening; (c) access to a smartphone; and (d) willingness to participate in either an AI-based digital intervention or a group-based support program. Exclusion criteria comprised current psychosis, acute suicidality, ongoing psychotherapy or psychiatric medication, and severe psychiatric conditions requiring immediate care.

At screening, participants completed an online consent form and eligibility questionnaires, including basic demographic and psychological distress assessments. Study staff contacted potential participants by phone or email to clarify missing or ambiguous data. After screening data were reviewed, eligibility was confirmed, and baseline assessments were completed, participants were randomly allocated in equal proportions (1:1:1) to one of three groups: AI intervention, group therapy, or waitlist control, using computer-generated block randomization stratified by sex. The randomization procedure was implemented by an independent research assistant who was not otherwise involved in the study and had no affiliation with the AI platform company. Allocation was concealed through the trial management platform, and data analysts were blinded to group assignment.

An a priori power analysis using G*Power 3.1 indicated that a total sample of 600 participants (approximately 200 per group) was required to detect a small-to-medium within–between interaction effect (*f*(*V*) = 0.10, *α* = 0.05, power = 0.80) in a 3 × 3 repeated-measures MANOVA. A larger sample was recruited to ensure sufficient power and account for potential attrition.

At baseline, participants provided informed consent, completed psychological questionnaires, and received an orientation regarding study procedures. Data collection began on April 10, 2025. Assessments were conducted at three time points: baseline (T1), immediately post-intervention following 12 weeks (T2), and at a 3-month follow-up (T3). Data were collected online through secure survey software. Participants received academic credit as compensation, and all provided informed consent before randomization. The study protocol was approved by the Institutional Review Board of the Baruch Ivcher School of Psychology, Reichman University (P_2024178), registered at ISRCTN (ISRCTN61075527; registration date: 12 May 2025), and conducted in accordance with the Declaration of Helsinki.

### AI-based digital intervention

Participants randomized to the digital intervention arm utilized Kai, a mobile and web-based AI platform developed by Kai.AI Inc. (Dover, Delaware, USA). Kai is an AI-powered emotional support system designed to simulate empathic, human-like dialogue and provide scalable, personalized psychological care. The platform operates directly within WhatsApp or iMessage, minimizing barriers to access and allowing participants to engage through a familiar messaging environment. In addition to its structured components, the AI platform provides open, supportive therapeutic dialogue that adaptively integrates cognitive-behavioral, mindfulness-based, and relational techniques according to participant needs.

To support these capabilities, the platform employs a multi-engine architecture that includes emotion-detection, intent-classification, open-dialogue generation, and structured-conversation engines. The system constructs a dynamic user profile based on information provided by users, clinician-supplied data when available, and conversational signals accumulated over time. The platform was developed and evaluated using clinician-designed simulations and synthetically generated training data rather than real therapeutic conversations, ensuring privacy-preserving model development. These engines draw on a research-based library of evidence-supported therapeutic protocols and exercises and guide response generation through a combination of specific fine-tuning, controlled prompting, and retrieval-augmented generation.

As part of onboarding, participants completed an initial assessment evaluating their emotional state, coping difficulties, and therapeutic preferences. These data were processed through an algorithmic decision system to generate an individualized support plan integrating personalization with evidence-based therapeutic content. The plan primarily comprised structured protocols targeting stress, anxiety, depressive symptoms, and other common emotional difficulties, supplemented by psychoeducation, breathing and relaxation exercises, brief mindfulness and meditation modules, and positive psychology tasks. Supportive affirmations and inspirational quotations were included to enhance engagement and motivation.

**Fig. 5:**
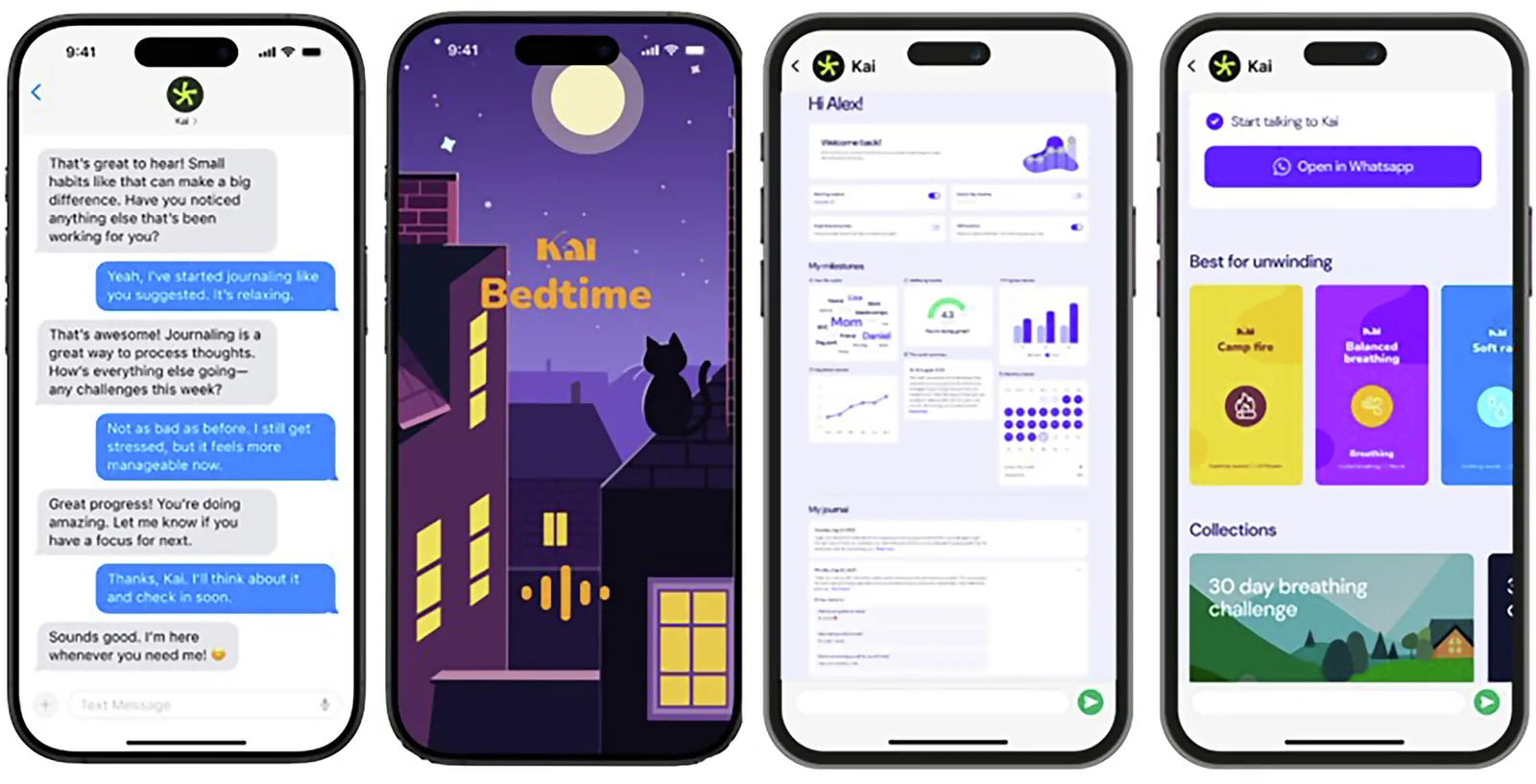
Sample screens from the Kai conversational AI platform*.* From left to right, the panels show an interactive text based dialogue providing empathic support and coping guidance, a video interface promoting sleep hygiene and relaxation, a personal journal summarizing user reflections and progress, and a content library offering guided exercises.

Participants also engaged in structured daily routines designed to promote emotional regulation and self-reflection. Morning sessions delivered supportive prompts and affirmations aimed at fostering adaptive coping, intention setting, and emotional preparedness. Evening sessions incorporated guided reflection to help participants review daily experiences, identify effective coping strategies, and acknowledge progress or positive emotional moments. These routines were intended to facilitate the integration of therapeutic principles into daily functioning and to reinforce self-awareness and resilience. A detailed description of intervention components is provided in Supplementary Table 4, and representative screenshots of the digital platform are presented in Fig. 5.

Participants were encouraged to engage with the conversational platform at least three times per week, with unrestricted access available throughout the 12-week intervention period. Session duration was self-paced, ranging from brief check-ins to extended interactions based on participant needs and preferences. Automated reminders supported consistent engagement, and weekly progress summaries provided personalized feedback on activity patterns and symptom change. To facilitate reflection and continuity, the platform generated individualized journals summarizing user interactions, insights, and changes in psychological indicators over time. All multimedia materials, including psychoeducational modules and guided exercises, remained accessible throughout the study to enable ongoing practice and reinforcement of therapeutic skills.

Personalization was a core design feature of the intervention. The platform’s adaptive memory system monitored prior interactions to maintain therapeutic continuity across sessions and to tailor subsequent responses to individual needs. Based on user input and symptom patterns, the system delivered targeted interventions such as relaxation protocols for sleep difficulties, attentional training for concentration challenges, and guided journaling for emotional processing. This adaptive feedback loop was intended to enhance treatment relevance, user engagement, and overall clinical effectiveness.

A comprehensive safety framework was integrated into the platform. A real-time risk-detection algorithm continuously screened user input for linguistic markers of acute distress, such as suicidal ideation or severe hopelessness, and automatically provided region-specific crisis hotline information and referral resources when needed. Beyond crisis detection, the platform employed multiple safety layers, including real-time content filters and refusal rules for diagnostic or clinical-direction requests, to prevent inappropriate or unsafe outputs. High-risk or flagged interactions were reviewed in real time by licensed clinicians, who could initiate direct outreach when necessary, ensuring a robust human-in-the-loop safety mechanism. All user interactions were encrypted, confidential, and stored on secure servers in compliance with data-protection regulations, supporting a safety architecture that emphasizes clinical oversight, data security, and responsible AI governance.

### Group therapy

Participants in the group therapy arm attended twelve weekly sessions, each lasting 90 min and conducted in groups of approximately 20 individuals, led by a licensed clinical psychologist. The intervention followed a semi-structured format, balancing structured therapeutic modules with flexibility to address themes arising from participants’ shared experiences. Each session incorporated time for open sharing and supportive-expressive dialogue, alongside structured modules integrating cognitive-behavioral therapy (CBT), acceptance and commitment therapy (ACT), dialectical behavior therapy (DBT), mindfulness-based techniques, positive psychology, and dynamic group processes.

Core elements included psychoeducation on stress, anxiety, and coping; physiological regulation practices (e.g., progressive muscle relaxation, diaphragmatic breathing, grounding); cognitive restructuring exercises (e.g., identifying and challenging unhelpful thoughts); mindfulness and acceptance practices; and positive psychology strategies (e.g., gratitude journaling, strengths identification). Sessions also incorporated reflective writing to deepen self-awareness and facilitate group discussion. Interpersonal skill-building (e.g., assertive communication, enhancing social support) was emphasized to strengthen peer connectedness. A detailed description of the group intervention components is provided in Supplementary Table 5.

Adherence was tracked through attendance records. Participants who missed sessions received summary materials and optional exercises to maintain therapeutic continuity. Treatment fidelity was assessed using facilitator checklists and weekly supervision meetings. Fidelity ratings indicated that psychologists delivered more than 90% of the planned content on average, with high consistency across groups. Upon completion of the intervention, participants completed a structured user experience questionnaire evaluating satisfaction and perceived therapeutic benefit.

### Control group

Participants in the control arm were assigned to a waitlist condition and did not receive any intervention during the 12-week trial period. They completed the same assessment battery as the intervention groups at baseline, post-intervention, and 3-month follow-up. To minimize attrition, control participants received periodic reminders to complete assessments and were granted academic credit equivalent to that of the intervention arms. Following completion of the final follow-up, they were offered access to their preferred intervention (either the AI-based program or group therapy).

### Psychological evaluation questionnaires

Changes in psychological states were assessed using validated self-report questionnaires administered at baseline, immediately post-intervention, and at 3-month follow-up. The measures included demographics and standardized instruments for depression (PHQ-9)^20^, generalized anxiety (GAD-7)^21^, PTSD symptoms (PCL-5 Short Form)^22^, life satisfaction (BMSLSS)^43^, and well-being (WHO-5)^44^. Individual differences were measured using validated scales for attachment style (ECR-R Short Form)^45^, perceived social support (MSPSS)^46^, and loneliness (UCLA Loneliness Scale Short Form)^47^.

### Application adherence and user experience

Adherence to the AI platform was evaluated through engagement metrics, including the number of active days during the intervention period and the average number of daily messages exchanged. Group therapy adherence was assessed using attendance records across the 12 sessions. At the end of the intervention, participants in both the AI and group therapy conditions completed a user experience questionnaire that assessed satisfaction, perceived usefulness, and ease of use. Open-ended responses allowed participants to comment on strengths, limitations, and suggestions for improvement.

### Statistical analysis

All statistical analyses were conducted using IBM SPSS Statistics 28. Descriptive statistics were calculated for demographic and baseline variables. Baseline differences between groups were examined with one-way ANOVAs and chi-square tests. Primary outcomes were analyzed using repeated-measures MANCOVA, with age and gender entered as covariates. When a significant multivariate condition × time interaction was detected, follow-up univariate ANCOVAs were performed with Bonferroni correction. Effect sizes are reported as partial *η*² and Cohen’s *d*. At the three-month follow-up, one-way MANCOVAs controlling for baseline measures, age, and gender were conducted. Moderation effects of loneliness, perceived social support, and attachment orientations were tested using the PROCESS macro (Model 1)^25^. In addition, a mediation analysis in AMOS 28 tested whether participant engagement with the AI-based intervention mediated the association between relational vulnerability factors and reductions in generalized anxiety symptoms. Model fit was evaluated using *χ*², CFI, TLI, SRMR and RMSEA indices.

## Supplementary information

Supplementary Information

## Data Availability

The datasets generated and analyzed during the current study are not publicly available due to privacy and ethical restrictions related to sensitive mental health data but are available from the corresponding author on reasonable request.
