# Supplementary material for: Attachment, loneliness, and social support as moderators of conversational AI–based mental health outcomes
Source: NPJ Digit Med. 2026 Jul 7;9:826. doi: 10.1038/s41746-026-02974-y (PMC13720182; doi:10.1038/s41746-026-02974-y)
Supplement: Supplementary file 1 [file 41746_2026_2974_MOESM1_ESM.pdf]

Attachment, Loneliness, and Social Support as Moderators of Conversational AI–Based Mental Health Outcomes

Anat Shoshani, Bar Gurfinkel, Ariel Kor , Or Kanarek , Romi Segev , Or Shafir , Romi Arbel ,
Yael Ben-Haim

Supplementary Information

1. Supplementary Tables

Supplementary Table 1. Relational factors moderating the effects of the ai intervention on mental health outcome changes ……………………..……………………..….……..……..1

Supplementary Table 2. Relational factors moderating the effects of the group intervention on mental health outcome changes ……...……………………………………………..……2

Supplementary Table 3. Regression coefficients for associations of baseline symptoms, relational vulnerabilities, and AI-intervention engagement with mental-health and well-being outcomes…………………………………………………………………………….....3

Supplementary Table 4. Structure and components of the AI–based digital psychological intervention………………………………………………………………………...…..5

Supplementary Table 5. Components of the group intervention (session modules and skills)……………………………………………………………………..………….7

2. Supplementary Figures

Supplementary Figure 1. CONSORT flow diagram………………………………………..9

Supplementary Figure 2. Relational factors, kai engagement, and anxiety outcomes.…10

Supplementary Table 1

Relational Factors Moderating the Effects of the AI Intervention on Mental Health Outcome Changes

Variables	Anxiety			Depression			PTSD			Well-being			Life Satisfaction		
	β	SE	*t*	*β*	SE	*t*	*β*	SE	*t*	*β*	SE	*t*	*β*	SE	*t*
**Loneliness Models**															
Constant	.02	.03	0.63	.01	.03	0.23	.01	.03	-0.11	-.01	.03	-0.32	.01	.03	0.06
AI intervention	-.33***	.03	-11.46	-.17***	.03	-5.30	-.02	.03	-0.67	.20***	.03	6.46	.15***	.03	4.88
Loneliness	-.09**	.03	-3.18	-.02	.03	-0.66	-.05	.03	-1.69	.02	.03	0.52	.09**	.03	2.90
AI intervention × Loneliness	-.15***	.03	-5.23	-.12***	.03	-3.82	-.01	.03	-0.36	.06*	.03	2.10	-.02	.03	-0.80
R^2	.16***			.05***			.01			.05***			.03***		
**Social Support Models**															
Constant	.01	.03	0.43	.01	.03	0.04	-.01	.03	-0.06	-.01	.03	-0.23	-.01	.03	-0.05
AI intervention	-.34***	.03	-11.50	-.17***	.03	-5.38	-.02	.03	-0.69	.20***	.03	6.46	.16***	.03	4.92
Social Support	.06	.04	1.82	.05	.04	1.27	.03	.04	0.91	-.11**	.04	-3.03	-.04	.04	-0.99
AI intervention × Social Support	.10*	.04	2.26	.02	.05	0.44	.07	.05	1.47	-.03	.05	-0.63	-.06	.05	-1.33
R^2	.13***			.03***			.01		¤	.05***			.03***		
**Avoidant Attachment Models**															
Constant	.01	.03	0.35	.01	.03	0.03	-.01	.03	-0.13	.01	.03	-0.21	.01	.03	0.01
AI intervention	-.34***	.03	-11.63	-.17***	.03	-5.43	-.02	.03	-0.77	.21***	.03	6.56	.16***	.03	5.01
Avoidant Attachment	-.05	.03	-1.62	-.02	.03	-0.73	.01	.03	0.34	.01	.03	0.27	.05	.03	1.45
AI intervention × Avoidant Attachment	-.07*	.03	-2.23	-.04	.03	-1.32	-.02	.03	-0.69	.03	.03	0.99	-.01	.03	-0.26
R^2	.13***			.03***			.01			.04***			.03***		
**Anxious Attachment Models**															
Constant	.01	.03	0.36	.01	.03	0.03	-.01	.03	-0.11	-.01	.03	-0.21	.01	.03	0.01
AI intervention	-.34***	.03	-11.67	-.17***	.03	-5.44	-.02	.03	-0.76	.21***	.03	6.56	.16***	.03	5.01
Anxious Attachment	-.07*	.03	-2.32	-.07*	.03	-2.37	-.03	.03	-1.01	.04	.03	1.30	.01	.03	0.04
AI intervention × Anxious Attachment	-.09**	.03	-2.91	-.02	.03	-0.72	-.04	.03	-1.16	.01	.03	0.16	.01	.03	0.44
R^2	.13***			.04***			.01			.04***			.03***		

Note: All outcome variables represent change scores (T2-T1). For anxiety, depression, and PTSD, negative beta values indicate symptom reduction. For well-being and life satisfaction, positive values indicate improvement in the measures. *$p < .05$, **$p < .01$, ***$p < .001$

Supplementary Table 2

Relational Factors Moderating the Effects of the Group Intervention on Mental Health Outcome Changes

**Variables**	**Anxiety**			**Depression**			**Well-being**		
	β	SE	*t*	*β*	SE	*t*	*β*	SE	*t*
**Loneliness Models**									
Constant	.01	.03	0.32	.01	.03	0.02	-.01	.03	-0.20
Group Therapy	.18***	.03	5.73	-.04	.03	-1.22	-.03	.03	-1.07
Loneliness	-.12***	.03	-3.85	-.04	.03	-1.12	.03	.03	0.91
Group Therapy × Loneliness	.11***	.03	3.47	.07*	.03	2.08	-.08**	.03	-2.61
R^2	.06***			. 01			.01*		
**Social Support Models**									
Constant	.01	.03	0.34	.01	.03	0.01	-.01	.03	-0.17
Group Therapy	.18***	.03	5.65	-.04	.03	-1.26	-.03	.03	-0.99
Social Support	.01	.04	0.38	.06	.04	1.64	-.15***	.04	-4.14
Group Therapy × Social Support	-.04	.04	-0.93	.04	.04	0.85	-.11*	.04	-2.49
R^2	.03***			.01			.02***		

Note: All outcome variables represent change scores (T2-T1). *$p < .05$, **$p < .01$, ***$p < .001$

Supplementary Table 3

Regression Coefficients for Associations of Baseline Symptoms, Relational Vulnerabilities, And AI-Intervention Engagement with Mental-Health and Well-Being Outcomes

Outcome	Predictor	B (SE)	β	t	p-value	95% CI
**Anxiety** (GAD7-T2)	Constant	5.38 (1.82)	—	2.96	.003	[1.803, 8.961]
	Anxiety_T1	.55 (.04)	.58	12.43	<.001	[.464, .639]
	Loneliness	.25 (.14)	.11	1.75	.08	[-.031, .520]
	Social support	-.44 (.27)	-.09	-1.62	.11	[-.977, .095]
	Avoidant attachment	.002 (.18)	.001	.014	.99	[-.356, .361]
	Anxious attachment	-.03 (.18)	-.007	-.14	.89	[-.374, .323]
	Engagement	-.01 (.001)	-.39	-8.31	<.001	[-.014, -.009]
	$R^2 = .43$, $F(6, 322) = 40.55$, $p < .001$					
**Depression** (PHQ9-T2)	Constant	4.91 (1.95)	—	2.51	.012	[1.079, 8.746]
	Depression_T1	.70 (.05)	.65	14.87	<.001	[.608, .794]
	Loneliness	.17 (.15)	.06	1.09	.28	[-.135, .470]
	Social support	-.53 (.29)	-.09	-1.80	.07	[-1.099, .049]
	Avoidant attachment	-.05 (.20)	-.01	-.27	.79	[-.437, .331]
	Anxious attachment	-.03 (.19)	-.006	-.14	.89	[-.399, .345]
	Engagement	-.009 (.001)	-.27	-6.25	<.001	[-.012, -.006]
	$R^2 = .52$, $F(6, 322) = 58.53$, $p < .001$					
**PTSD** (PCL-5-T2)	Constant	.79 (1.40)	—	.57	.57	[-1.966, 3.551]
	PTSD_T1	.63 (.04)	.65	14.89	<.001	[.548, .715]
	Loneliness	.23 (.11)	.12	2.21	.03	[.026, .442]
	Social support	-.07 (.21)	-.02	-.33	.74	[-.487, .346]
	Avoidant attachment	.11 (.14)	.04	.79	.43	[-.164, .383]
	Anxious attachment	-.13 (.14)	-.04	-.92	.36	[-.390, .141]
	Engagement	-.004 (.001)	-.17	-4.02	<.001	[-.006, -.002]
	$R^2 = .500$, $F(6, 322) = 53.628$, $p < .001$					

**Life Satisfaction** (LS-T2)	Constant	10.306 (1.809)	—	5.70	<.001	[6.748, 13.864]
	LS_T1	.65 (.04)	.67	14.81	<.001	[.560, .732]
	Loneliness	-.08 (.12)	-.04	-.68	.49	[-.308, .149]
	Social support	.36 (.25)	.07	1.47	.14	[-.122, .840]
	Avoidant attachment	-.13 (.15)	-.04	-.88	.38	[-.434, .167]
	Anxious attachment	-.13 (.15)	-.04	-.85	.40	[-.418, .166]
	Engagement	.003 (.001)	.09	2.14	.03	[.000, .005]
	$R^2 = .57$, $F(6, 322) = 70.29$, $p < .001$					
**Well-Being** (WHO5-T2)	Constant	26.32 (7.79)	—	3.38	<.001	[10.995, 41.644]
	Well-being_T1	.63 (.05)	.59	12.41	<.001	[.527, .726]
	Loneliness	-.81 (.56)	-.09	-1.43	.15	[-1.916, .300]
	Social support	.51 (1.10)	.03	.46	.65	[-1.661, 2.677]
	Avoidant attachment	-.42 (.73)	-.03	-.57	.57	[-1.849, 1.019]
	Anxious attachment	-.13 (.71)	-.009	-.18	.86	[-1.518, 1.260]
	Engagement	.02 (.005)	.20	4.21	<.001	[.012, .034]
	$R^2 = .43$, $F(6, 322) = 39.94$, $p < .001$					

Note: AI intervention (n = 329).

Supplementary Table 4

Structure and Components of the Kai AI–based Digital Psychological Intervention

Component	Description	Therapeutic Focus
**1. Adaptive Onboarding and User Profiling**	The platform's adaptive onboarding process identifies user needs, emotional tendencies, coping styles, and personal goals through short questionnaires and conversational exchanges. This initial mapping allows the AI system to individualize tone, communication style, and therapeutic strategies, creating a supportive environment that reflects each user's unique psychological profile.	Individual tailoring, early engagement, and establishment of therapeutic relevance.
**2. Emotional Health Tracking and Check-ins**	Users complete brief daily or weekly check-ins and standardized self-report questionnaires assessing key domains such as anxiety, depression, stress, and well-being. Reflective prompts accompany these assessments, and aggregated data are visualized to reveal emotional patterns and progress over time, allowing early recognition of difficulties or improvements.	Emotional literacy, proactive self-observation, and maintenance of adaptive coping.
**3. Personalized Psychoeducational Library**	Personalized psychoeducation is provided through concise written and video-based materials that introduce evidence-based methods for managing thoughts, emotions, and behaviors. Topics include stress management, motivation, relationships, compassion, and self-regulation, among others. Content is dynamically adjusted according to user characteristics, current mood, and engagement trends.	Knowledge enhancement, normalization of experiences, and development of cognitive insight.
**4. Mindfulness and Skill-Building Exercises**	A diverse library of short, guided audio and video sessions offers mindfulness and breathing exercises, grounding techniques, brief meditations, and ACT, CBT and DBT-based coping practices. The AI recommends content based on current user data and emotional needs, fostering both momentary regulation and sustained psychological balance.	Regulation of affect, distress tolerance, and cultivation of mindful presence.
**5. Evidence-Based Therapeutic Frameworks**	Structured, evidence-based modules and protocols draw from CBT, ACT, DBT, mindfulness, compassion-focused, and positive psychology frameworks. The AI flexibly integrates these methods to address diverse psychological processes such as maladaptive thinking, avoidance, and emotional dysregulation, adapting to each user's evolving context.	Cognitive and emotional flexibility, improved problem-solving, and strengthening of coping skills.

**6. AI-Mediated Supportive Dialogue (Text/Voice)**	Users engage in natural text or voice dialogues that foster supportive and therapeutic communication. The AI employs clinical techniques such as empathic reflection, Socratic questioning, emotion labeling, behavioral activation, and values clarification, adapting responses in real time to tone, language, and emotional intensity.	Empathic resonance, collaborative meaning-making, and facilitation of adaptive perspectives.
**7. Reflective Journal and Insight Integration**	The platform continuously aggregates conversational and emotional data into a personal digital journal. Automated daily and weekly summaries highlight recurring themes, emotional shifts, and engagement trends, enabling users to consolidate insights, monitor growth, and strengthen self-awareness over time.	Self-reflection, consolidation of learning, and recognition of personal growth.
**8. Daily Well-Being Routines and Micro-Practices**	Morning, midday, and evening routines embed intention-setting, gratitude reflections, mindfulness breaks, and brief psychoeducational or coping exercises into daily life. Each session is concise and adaptive, designed to promote balance, awareness, and emotional resilience within natural routines.	Routine stabilization, sustained emotional equilibrium, and reinforcement of healthful habits.
**9. Growth-Focused and Positive Psychology Modules**	Longer sessions are dynamically guided by AI and evolve according to user responses, emotional states, and engagement patterns. Drawing on positive psychology and well-being science, these sessions explore meaning, personal strengths, gratitude, and value-based goal setting to promote resilience and enduring well-being.	Development of purpose, enhancement of positive affect, and long-term resilience building.
**10. Continuous Safety Monitoring and Clinical Response**	Advanced natural-language algorithms continuously analyze user communication for linguistic or emotional indicators of acute distress or potential risk (e.g., hopelessness, self-harm intent, or crisis expressions). When a concern is detected, a tiered safety protocol is activated that includes prompt review by licensed mental health professionals, provision of crisis-support resources, and, when necessary, direct outreach to the user or coordination with emergency services to ensure safety.	Risk detection, user safety assurance, and integration of ethical clinical oversight.

Note: All user communications are fully encrypted and stored securely in compliance with international privacy and data protection regulations. No human personnel have access to user conversations or personal data under normal circumstances. Limited and supervised access may occur only in confirmed risk situations, as part of the platform's safety protocol, to ensure user protection. These privacy and safety procedures are transparently explained to all users in the platform's terms of use.

Supplementary Table 5

The Psychological Group Intervention

Session	Name of Intervention	Explanation
**1**	Orientation and Emotional Health Foundations	The opening session establishes group norms, confidentiality, and emotional safety. Psychoeducation introduces the relationship between stress, emotion, and the body–mind system. A collaborative "coping pathway" exercise helps participants identify personal triggers and habitual responses. Open group sharing invites discussion of stress and coping experiences and expectations, fostering trust, empathy, and initial group cohesion.
**2**	Emotional, Behavioral, and Cognitive Change	This session emphasizes the process of change through activation and cognitive flexibility. Psychoeducation explains the interaction between behavior, thought, and emotion, highlighting how engaging in purposeful actions can increase energy, mood, and motivation. Participants practice identifying small, achievable steps that counter avoidance and support valued goals. Group sharing focuses on experiences of initiating action, managing emotional shifts, and recognizing the link between doing, thinking, and feeling.
**3**	Emotional Regulation and the Permission to Feel	This session focuses on developing emotional balance through relaxation, grounding, and mindful breathing. Psychoeducation addresses the connection between the body, breath, and emotions, emphasizing how awareness and acceptance support recovery and self-regulation. Participants practice guided relaxation, imagery, and diaphragmatic breathing to ease tension and restore calm. Incorporating DBT and acceptance-based approaches, the group explores ways to recognize and manage difficult emotions while maintaining stability. A key element involves allowing oneself to experience emotions freely—the permission to be human—without judgment or avoidance. Reflection and sharing center on experiences of stress and relief, moments of self-acceptance, and strategies for fostering a sense of safety, steadiness, and personal growth.
**4**	Cognitive Flexibility I – Identifying Thought and Emotion Patterns	Participants explore the relationship between automatic thoughts, emotional reactions, and behavior. Through guided dialogue and shared examples, they recognize common cognitive patterns such as catastrophizing or self-blame. Group sharing normalizes these experiences and deepens emotional insight through mutual understanding and validation.
**5**	Cognitive Flexibility II – Perspective Shifting and Adaptive Thinking	The focus is on reframing distressing thoughts and developing balanced perspectives. Participants reflect on personal situations and share moments of insight where compassion or evidence challenged rigid thinking. The group sharing emphasizes cognitive and emotional shifts as pathways to self-awareness and stability.
**6**	Mindfulness and Attention Training	Psychoeducation covers rumination and attention biases as contributors to emotional distress. Guided mindfulness practices (body scan, observation, and grounding) are followed by reflection on the experience of presence and

Session	Name of Intervention	Explanation
		self-acceptance. Participants share moments of calm, challenge, or discovery, linking mindfulness to emotional regulation and self-compassion.
**7**	Acceptance and Values-Based Action (ACT Principles)	This session focuses on acceptance and commitment principles, emphasizing openness to inner experience and action guided by personal values. Participants clarify what matters most to them and discuss how value-consistent behavior supports resilience, authenticity, and motivation. Group sharing explores difficulties in sustaining valued actions during distress and how alignment with core values can foster long-term emotional balance and flexibility.
**8**	Self-Compassion and Emotional Validation	This session focuses on reducing self-criticism and cultivating a compassionate inner stance. Participants engage in guided compassion exercises and expressive journaling to deepen emotional awareness. Group dialogue centers on experiences of extending kindness toward oneself and others, acknowledging vulnerability, and recognizing how shared empathy and acceptance can foster healing, confidence, and emotional resilience.
**9**	Gratitude, Savoring, and Hope	Participants learn how gratitude and positive emotions enhance coping and recovery. Writing tasks such as “three good things” or recalling meaningful experiences are followed by shared reflection. The group explores how collective expressions of appreciation and hope foster motivation, optimism, and a sense of connection.
**10**	Strengths, Growth, and Meaning	Participants identify core strengths that have supported them during periods of stress and difficulty. Through guided reflection and sharing, they explore how using these strengths fosters learning, personal growth, and meaning-making. Discussion emphasizes how awareness of strengths and purpose can enhance resilience, confidence, and sustained psychological health.
**11**	Relational Awareness and Healthy Boundaries	Psychoeducation introduces patterns of attachment, communication, and relational dynamics in emotional health. Participants are invited to reflect on their experiences of connection, distance, and support within relationships. Group sharing focuses on recognizing emotional needs, boundaries, and the role of authentic connection in recovery and well-being.
**12**	Integration, Continuity, and Closure	The final session consolidates therapeutic learning and emotional progress. Guided imagery and reflective writing encourage integration of insights and appreciation for personal change. Group dialogue supports mutual recognition, closure, and discussion of strategies to sustain growth and self-care beyond the intervention.

Note: This structured 12-session group therapy incorporates psychoeducation, experiential exercises, and guided group sharing to facilitate emotional insight, regulation, and personal growth. draws on principles from cognitive-behavioral therapy (CBT), acceptance and commitment therapy (ACT), dialectical behavior therapy (DBT), mindfulness-based interventions, and positive psychology

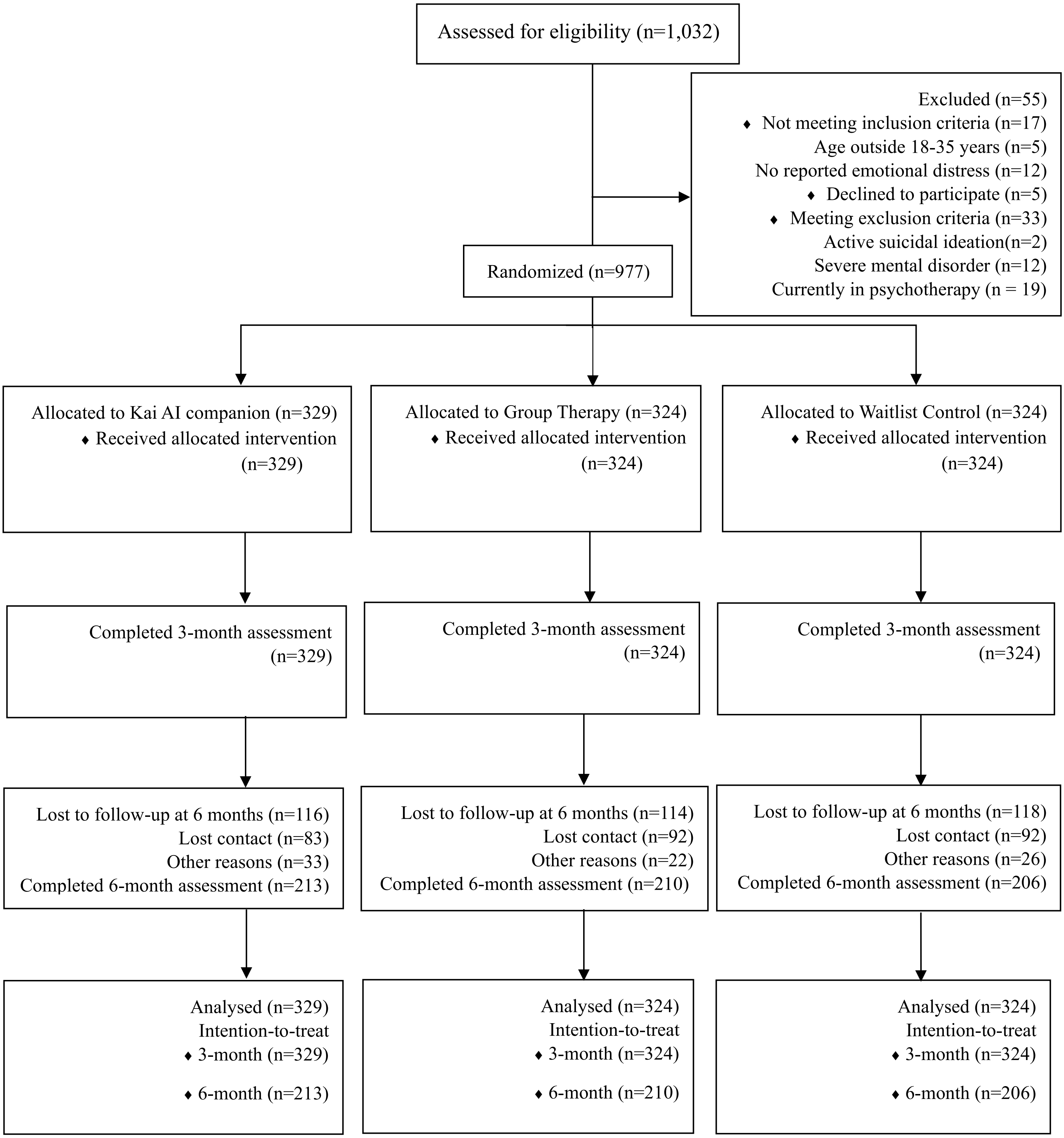

Supplementary Figure 1: CONSORT Flow Diagram

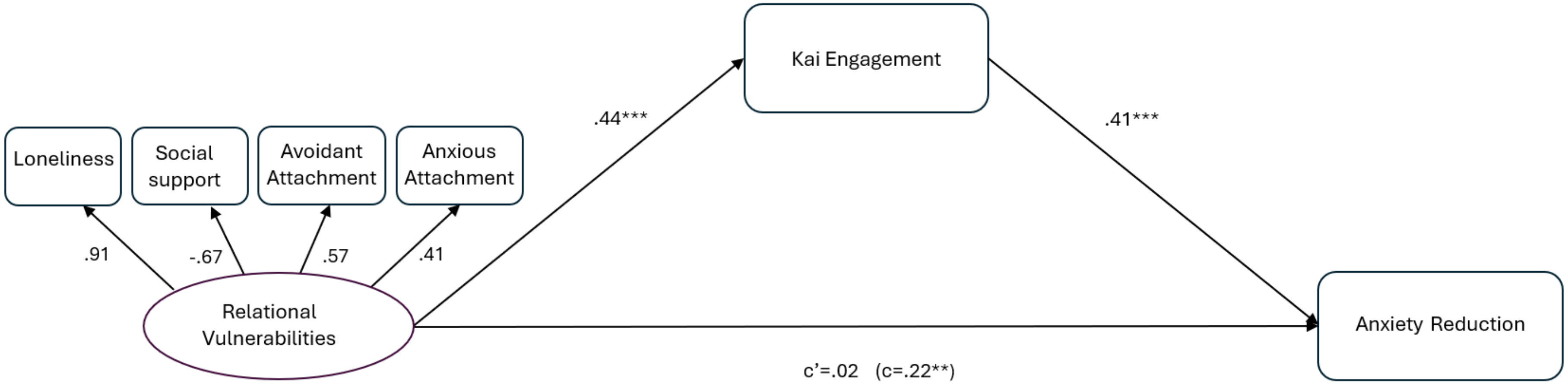

Supplementary Figure 2: Relational Factors, Kai Engagement, and Anxiety Outcomes. The diagram presents a structural equation model linking a latent relational vulnerability factor indexed by loneliness, perceived social support, avoidant attachment, and anxious attachment to engagement with the AI intervention and subsequent change in anxiety symptoms. Relational vulnerabilities were positively associated with greater engagement, which in turn predicted larger reductions in anxiety. When engagement was included in the model, the direct association between relational vulnerabilities and anxiety change was no longer significant, indicating full mediation by engagement. Path coefficients represent standardized estimates; significant paths are indicated.
